# Caldomycin, a new guanidopolyamine produced by a novel agmatine homocoupling enzyme involved in homospermidine biosynthesis

**DOI:** 10.1038/s41598-024-58296-0

**Published:** 2024-03-30

**Authors:** Teruyuki Kobayashi, Akihiko Sakamoto, Tamao Hisano, Keiko Kashiwagi, Kazuei Igarashi, Koichi Takao, Takeshi Uemura, Takemitsu Furuchi, Yoshiaki Sugita, Toshiyuki Moriya, Tairo Oshima, Yusuke Terui

**Affiliations:** 1 Faculty of Pharmacy, Chiba Institute of Science, 15-8 Shiomi-cho, Choshi, Chiba 288-0025 Japan; 2 Department of Pathophysiology and Therapeutics, Hoshi University School of Pharmacy and Pharmaceutical Sciences, Shinagawa, Tokyo Japan; 3 RIKEN Center for Biosystems Dynamics Research (BDR), Tsurumi, Kanagawa Japan; 4 Amine Pharma Research Institute, Innovation Plaza at Chiba University, Chiba, Japan; 5 Faculty of Pharmacy and Pharmaceutical Sciences, Josai University, Sakado, Saitama Japan; 6 Institute of Environmental Biology, Kyowa-Kako, Machida, Tokyo Japan; 7 School of Pharmacy, International University of Health and Welfare, Otawara, Tochigi Japan

**Keywords:** Enzymes, Bacteriology

## Abstract

An extreme thermophilic bacterium, *Thermus thermophilus* produces more than 20 unusual polyamines, but their biosynthetic pathways, including homospermidine, are not yet fully understood. Two types of homospermidine synthases have been identified in plants and bacteria, which use spermidine and putrescine or two molecules of putrescine as substrates. However, homospermidine synthases with such substrate specificity have not been identified in *T. thermophilus*. Here we identified a novel agmatine homocoupling enzyme that is involved in homospermidine biosynthesis in *T. thermophilus*. The reaction mechanism is different from that of a previously described homospermidine synthase, and involves conjugation of two molecules of agmatine, which produces a diamidino derivative of homospermidine (caldomycin) as an immediate precursor of homospermidine. We conclude that there is a homospermidine biosynthetic pathway from agmatine via caldomycin synthase followed by ureohydrolase in *T. thermophilus*. Furthermore, it is shown that caldomycin is a novel compound existing in nature.

## Introduction

The major polyamines putrescine, spermidine and spermine are found in eukaryotes, bacteria, and archaea^1^, and are essential for cell growth and viability^2–4^. Their major functions include interactions with nucleic acids and effects on RNA translation^5–8^. In higher plants, polyamines are involved in the regulation of diverse physiological functions such as organogenesis, embryogenesis, flowering and fruit maturation^9,10^. In addition, polyamines that covalently combine with a phenolic compound are involved in the synthesis of pyrrolizidine alkaloids. Pyrrolizidine alkaloids are produced by plants as defense compounds against herbivory by insects, but they are also toxic to humans and can lead to liver failure and carcinogenesis^11–13^.

**Figure 1:**
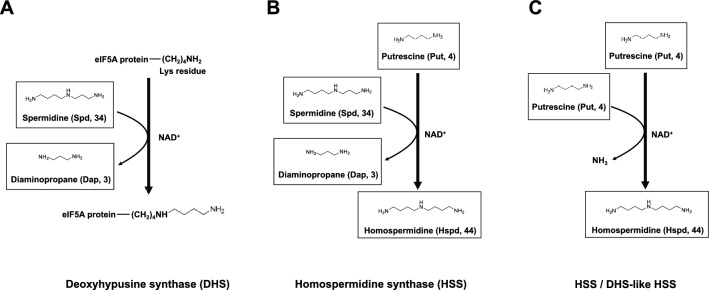
Enzymatic reactions catalyzed by human deoxyhypusine synthase (DHS) (**A**) and two types of homospermidine synthase (HSS) in *Arabidopsis thaliana* (**B**) and *Senecio vernalis* and *Cylindrospermum stagnale* (**C**) in homospermidine biosynthesis. These enzymes transfer an aminobutyl group of putrescine or spermidine to their individual substrates in an NAD^+^-dependent reaction. DHS catalyses the first rate-limiting step of a unique post-translational modification: hypusination. This modification occurs exclusively on one protein, eukaryotic translation initiation factor 5A (eIF5A), and it is essential for cell proliferation^28^.

Homospermidine synthesized by homospermidine synthase (HSS) is an essential intermediate for biosynthesis of pyrrolizidine alkaloids in plants^14,15^, and only one HSS involved in their biosynthesis has been identified to date. Two types of HSS have been identified in plants and bacteria (Fig. 1)^14,16^. The first type of HSS catalyzes the synthesis of homospermidine from putrescine and spermidine (Fig. 1B)^14^, similar to eukaryotic deoxyhypusine synthase (DHS) (Fig. 1A). The second type of HSS catalyzes a condensation reaction of two molecules of putrescine (Fig. 1C)^16–18^. This type of reaction is found widely in α-proteobacteria^19^.

Bacteria, archaea and algae produce unusual polyamines such as long-chain and branched-chain polyamines^20,21^ that are essential for cell growth and viability in extreme environments^22–24^. An extreme thermophile, *Thermus thermophilus* produces more than 20 unusual polyamines containing propyl or butyl groups, and their metabolic pathways and synthases are unique^25,26^. *T. thermophilus* also produces homospermidine, and we have previously reported that triamine/agmatine aminopropyltransferase (TtSpeE/TAAPT) gene-disrupted strain, which is an important enzyme for the production of polyamines containing an aminopropyl moiety, produces homospermidine as the major component^24^. Therefore, it is thought that homospermidine is an alternative polyamine for cell growth and viability under extreme environments. In fact, homospermidine stimulates cell growth of *Escherichia coli* at 42 °C effectively compared with other triamines^27^. However, the biosynthetic pathway for homospermidine biosynthesis in *T. thermophilus* is not fully understood.

Here we show that a DHS gene homolog (TTHA1570) in *T. thermophilus* is a key enzyme involved in the synthesis of homospermidine. An assay of the purified enzyme showed that it utilizes agmatine as the acceptor in its agmatinyl group transfer reaction. The enzymatic product is a conjugation of two molecules of agmatine, which produces a diamidino derivative of homospermidine (caldomycin, Cdm). Therefore, the enzyme was named caldomycin synthase (CdmS). This is the first report of a polyamine synthase responsible for the transfer of an agmatinyl group but whose reaction mechanism is different from HSS and DHS-like HSS.

## Results and Discussion

### The reaction mechanism of CdmS (caldomycin synthase) differs from that of exiting homospermidine synthase

We have previously reported that homospermidine is produced as the major polyamine in a gene-disrupted strain for the triamine/agmatine aminopropyl transferase (TtSpeE/TAAPT), a key enzyme for the production of polyamines possessing an aminopropyl moiety in *T. thermophilus*^24^. Therefore, it is possible that this bacterium possesses a homospermidine biosynthetic pathway different from the pathways for synthesis of other long and branched polyamines. The DHS gene homolog (TTHA1570) was found in the *T. thermophilus* genome. It is known that the functional properties of HSS are similar to that of DHS (Fig. 1A), although the number of amino acids and substrate recognition varies among organisms. Thus, the gene of DHS homolog which is the putative polyamine biosynthetic enzyme is named as *cdmS*. We generated an alignment of the highly conserved HSS, DHS and DHS-like HSS (SpeY) amino acid sequences from *T. thermophilus*, human, *Arabidopsis thaliana*, *Cylindrospermum stagnale* and *Senecio vernalis* using Clustal Omega (Supplemental Fig. 1). The sequences of CdmS showed 27 to 33% identity with the enzymes of other organisms. The lysine residue that is critical for the catalytic activity of DHS is conserved in these proteins^28^.

**Figure 2:**
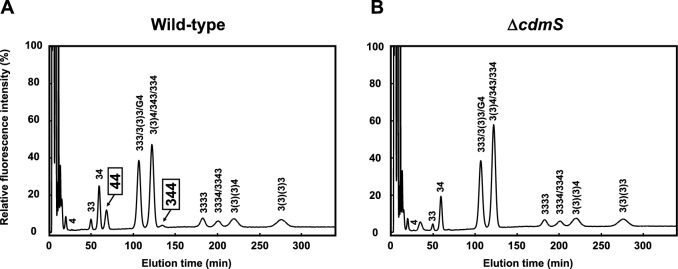
HPLC chromatograms of polyamines in *T. thermophilus* HB8 (**A**) and ∆*cdmS* (**B**) strains grown at 70 °C. Polyamine composition in cells harvested at an A_600_ of 0.5 was measured as described in Materials and Methods. 4, putrescine; 33, norspermidine; 34, spermidine; 44, homospermidine; G4, agmatine; 333, thermine; 3(3)3, mitsubishine; 343, spermine; 334, thermospermine; 3(3)4, *N*^4^-aminopropylspermidine; 344, homospermine; 3333, caldopentamine, 3334, homocaldopentamine; 3343, thermopentamine; 3(3)(3)4, *N*^4^-bis(aminopropyl)spermidine; 3(3)(3)3, tetrakis(3-aminopropyl)ammonium. The abbreviation numbering scheme reflects the number of carbon atoms separating the amino or aza groups in each molecule.

To investigate whether CdmS is involved in homospermidine biosynthesis, we constructed a *cdmS* gene-disrupted strain of *T. thermophilus.* To eliminate the influence of polyamines present in the culture medium components, *T. thermophilus* was grown in minimum medium and the intracellular polyamine content of the wild-type and gene-disrupted (Δ*cdmS*) strains was analyzed by HPLC (Fig. 2). Peaks corresponding to homospermidine (44) and homospermine (344) were undetectable in the Δ*cdmS* strain. This suggests that CdmS is essential for synthesis of homospermidine, and that homospermine (344) is synthesized from homospermidine (44) but not from spermidine (34).

**Figure 3:**
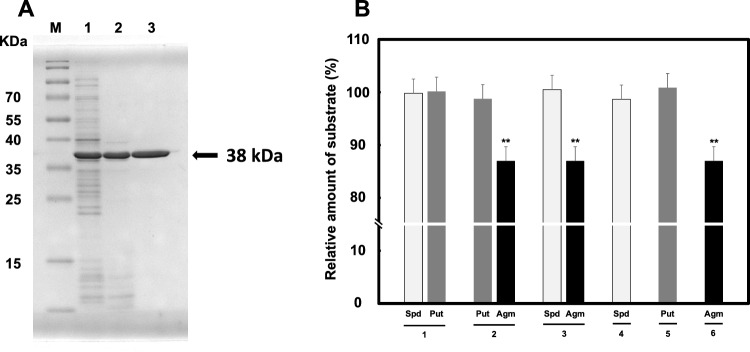
(**A**) SDS-PAGE analysis of purified CdmS. Protein bands were stained with Coomassie Brilliant Blue R-250. Lane M, protein marker Broad Range (Thermo Scientific). Lane 1, *E. coli* BL21(DE3) crude extract after induction with 1 mM IPTG. Lane 2, heat treatment. Lane 3, CdmS purified by TOYOPEARL Butyl 650 M column. The original gel is presented in Supplemental Fig. 4. (**B**) CdmS activity toward putative substrate polyamines. Lanes 1, substrates are spermidine and putrescine. Lanes 2, substrates are putrescine and agmatine. Lanes 3, substrates are spermidine and agmatine. Lanes 4, substrate is spermidine. Lanes 5, substrate is putrescine. Lanes 6, substrate is agmatine. *Spd* spermidine; *Pu*, putrescine; *Agm* agmatine. The results are mean ± standard error of triplicate determinations. Student’s *t*-test was performed for the value of obtained in the presence of CdmS versus its absence. **, *p* < 0.01.

CdmS was purified to determine its enzymatic properties. As shown in Fig. 3A, a single prominent band with an apparent molecular mass of 38 kDa was obtained by SDS-PAGE. The enzymatic activity of CdmS was studied by adding possible substrate polyamines putrescine, spermidine, or agmatine and combinations (Fig. 3B). Surprisingly, CdmS did not affect levels of putrescine and spermidine (Fig. 3B, *lanes 1*, *4* and *5*) but reduced the level of agmatine (Fig. 3B, *lanes 2*,* 3* and *6*) suggesting that agmatine was used as the sole substrate. However, in these assays when agmatine was included as the substrate for CdmS, homospermidine was never detected as a product. Thus, if CdmS is involved in homospermidine synthesis in vitro, there is presumably an intermediary product between agmatine and homospermidine, synthesized by CdmS that serves as the substrate for synthesis of homospermidine.

### Identification of reaction product by CdmS

**Figure 4:**
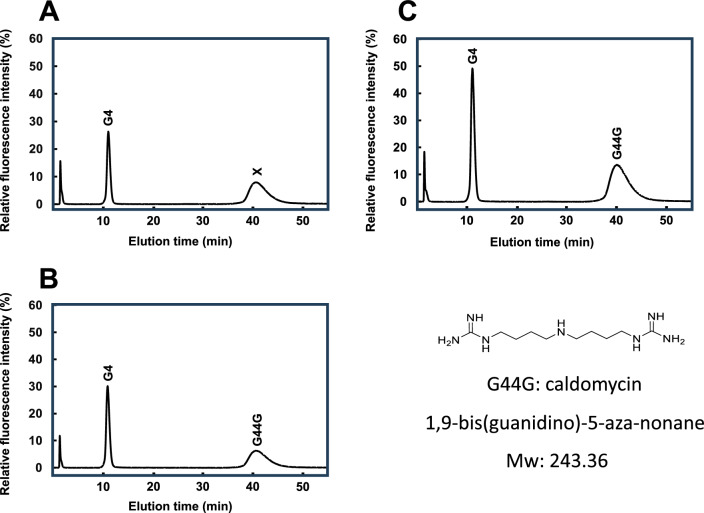
HPLC chromatograms of guanidyl derivative analysis. The reaction mixture was separated by HPLC benzoin-couple products detected by fluorescence. (**A**) In vitro polyamine synthesis using purified CdmS after agmatine (G4) was incubated with CdmS. X, unknown peak. (**B**) Peak standard using synthetic G44G. (**C**) Mixture of enzymatic reaction mixture and synthetic G44G.

Agmatine was the only identified substrate for CdmS. The results suggest that the first step in homospermidine biosynthesis is a condensation reaction of agmatine. Such a reaction would produce an agmatine derivative, 1,9-bis(guanidino)-5-aza-nonane. However, this guanidyl compound cannot react with *o*-phthalaldehyde and cannot be detected using the same HPLC method used to identify polyamines and polyamine derivatives such as spermine, spermidine, and homospermidine. Thus, a different fluorescent reagent, benzoin^29^, which interacts with guanidyl compounds, was used to try to detect 1,9-bis(guanidino)-5-aza-nonane after HPLC separation of the reaction products. As shown in Fig. 4A, peaks corresponding to agmatine (G4) and the putative product of CdmS, “X” were observed after incubation of agmatine with CdmS. The product X is likely 1,9-bis(guanidino)-5-aza-nonane formed via agmatine coupling. To determine if this is the case, synthetic 1,9-bis(guanidino)-5-aza-nonane was compared with product X by HPLC. As shown in Fig. 4B, synthetic 1,9-bis(guanidino)-5-aza-nonane eluted with the same retention time as product X. When the enzymatic reaction included synthetic 1,9-bis(guanidino)-5-aza-nonane as well as the substrate agmatine, the peak area of product X was increased but the retention time was unchanged (Fig. 4C). The results indicate that product X was indeed 1,9-bis(guanidino)-5-aza-nonane, and is named as caldomycin (Cdm). This is the first report to show the natural occurrence of caldomycin.

### New homospermidine biosynthesis from agmatine by caldomycin synthase (CdmS) together with aminopropylagmatine ureohydrolase (TtSpeB_ap_) in *T*. *thermophilus*

**Figure 5:**
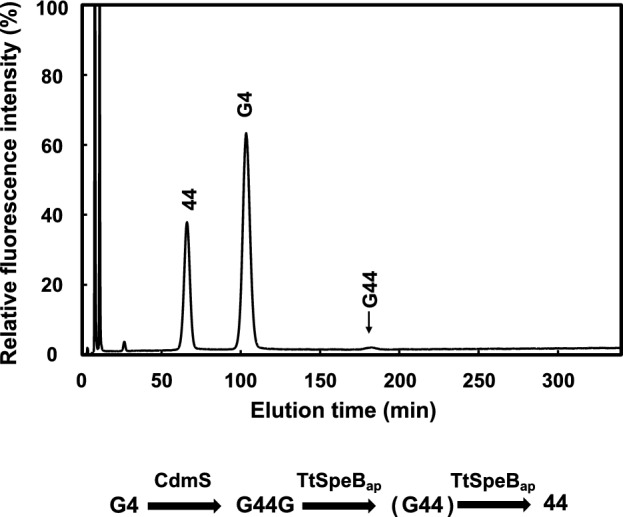
In vitro polyamine syntheses using purified CdmS and TtSpeB_ap_. The HPLC chromatogram shows polyamine composition after agmatine (G4) was incubated with CdmS and TtSpeB_ap_. G44, *N*^1^-aminobutylagmatine; 44, homospermidine.

To confirm that CdmS converts agmatine to caldomycin and that TtSpeB_ap_ uses caldomycin as a substrate to produce homospermidine, we purified CdmS and TtSpeB_ap_ and carried out in vitro enzymatic reactions followed by HPLC analysis. As shown in Fig. 5, the combination of CdmS and TtSpeB_ap_ with agmatine (G4) produced homospermidine (44). In addition, a new intermediate compound was detected as a minor component. The elution time suggested that the new compound is *N*^1^-aminobutylagmatine (G44). The structure of the intermediate was confirmed by comparing its elution time with synthetic *N*^1^-aminobutylagmatine.

The enzymatic activities of CdmS for substrates such as agmatine, diaminopropane, putrescine, norspermidine, and spermidine revealed that the enzyme has transferase activity. These results indicated that CdmS utilizes agmatine as the only acceptor in its agmatinyl group transfer reaction (*K*_m_; 0.095 ± 0.006 mM, *V*_max_; 1.07 ± 0.05 µmol/min/mg). Therefore, the enzyme was named as caldomycin synthase (CdmS). In addition, the enzymatic activities of TtSpeB_ap_ and TtARG for caldomycin revealed that the two enzymes have ureohydrolase activity as shown in Table 1. In particular, TtSpeB_ap_ has very high ureohydrolase activity against caldomycin. In in vivo studies of *T. thermophilus*, caldomycin and *N*^1^-aminobutylagmatine were undetectable, suggesting that TtSpeB_ap_ rapidly converts caldomycin to homospermidine in vivo.

**Table 1:** Kinetic parameters of ureohydrolases.

Enzyme	Substrate	*K*_m_ (mM)	*V*_max_ (µmol/min/mg)	*k*_cat_ (s^−1^)	*k*_cat/_*K*_m_ (M^−1^ s^−1^)
TtSpeB_ap_	Caldomycin	3.84 ± 0.84	2920 ± 420	1580 ± 230	419,000 ± 32,000
TtARG	Caldomycin	9.00 ± 3.00	0.0105 ± 0.019	0.0055 ± 0.0010	0.64 ± 0.10

The rate of the reaction for CdmS in the presence of a smaller amount of TtSpeB_ap_ was significantly increased by temperature elevation from 40 to 65 °C (Supplemental Fig. 2A). The effect of pH on enzymatic activity was investigated over pH range of 6.0 to 10.5 at 65 °C (Supplemental Fig. 2B). CdmS showed highest activity at around pH 8.5. We previously reported that homospermidine is increased in *T. thermophilus* grown at relatively lower temperature such as 60 °C, but there is a minor component in the cells grown at the optimal growth temperature such as 70 °C. In addition, cell growth and polyamine contents of the polyamine-deficient strain (Δ*TtspeA*) were recovered when agmatine was added to the culture medium at 60 °C^23^. Especially, homospermidine levels increased together with homospermine. These results indicate that there is a good correlation between the substrate and temperature at which CdmS acts efficiently and the polyamine composition in *T. thermophilus*.

### Synthetic data are summarized in the following points:

- 1,9-bis(guanidino)-5-aza-nonane (caldomycin, G44G);

^1^H NMR (D_2_O, 400 MHz) δ 1.62–1.82 (8H, m, 2 × CH_2_CH_2_CH_2_CH_2_), 3.07 (4H, t, *J* = 7.6 Hz, CH_2_NCH_2_), 3.23 (4H, t, *J* = 6.7 Hz, 2 × CH_2_NHC(NH)NH_2_). ^13^C NMR (D_2_O, 100 MHz) δ 22.79, 25.01 (2 × CH_2_CH_2_CH_2_CH_2_), 40.39 (CH_2_NCH_2_), 47.03 (2 × CH_2_NHC(NH)NH_2_), 156.78 (2 × CH_2_NHC(NH)NH_2_). HR-MS (FAB) m/z: 244 [M + H]^+^. Calcd for C_10_H_24_N_7_ 244.2250, Found 244.2256.

- 1-guanidino-9-amino-5-azanonane (*N*^1^-aminobutylagmatine, G44);

^1^H NMR (D_2_O, 400 MHz) δ 1.62–1.85 (8H, m, 2 × CH_2_CH_2_CH_2_CH_2_), 3.00–3.15 (6H, m, 3 × NCH_2_), 3.25 (2H, t, *J* = 6.8 Hz, CH_2_NHC(NH)NH_2_). ^13^C NMR (D_2_O, 100 MHz) δ 22.73, 22.84, 23.92, 25.09 (2 × CH_2_CH_2_CH_2_CH_2_), 38.79, 40.46 (CH_2_NCH_2_), 46.84, 47.12 (2 × NCH_2_), 156.75 (CH_2_NHC(NH)NH_2_). HR-MS (FAB^+^) m/z: 202 [M + H]^+^. Calcd for C_9_H_24_N_5_ 202.2032, Found 202.2031.

### The catalytic mechanism of CdmS is highly homologous to that of the human DHS

**Figure 6:**
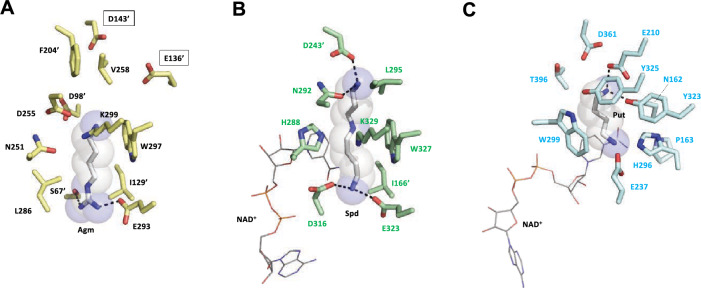
Substrate-binding sites of (**A**) CdmS from *T. thermophilus*, (**B**) DHS from human (HsDHS) (PDB ID 6XXJ), and (**C**) HSS from *Blastochloris viridis* (BvHSS) (PDB ID 4TVB). Residue numbers with primes (ʹ) indicate the residues are from the neighboring subunit. Possible polar interactions between residues and ligands are indicated by broken lines. (**A**) A docking model of the agmatine (Agm) molecule is shown with transparent spheres. The residue numbers of putative guanidyl group-binding residues for a second agmatine substrate are boxed. (**B**, **C**) The bound spermidine (Spd) and putrescine (Put) molecules are shown with transparent spheres. The bound NAD molecule is shown as a line model.

The structures of human DHS (HsDHS)^30^ and *Blastochloris viridis* HSS (BvHSS)^31^ have been proposed based on X ray crystallography, providing insights into their catalytic mechanisms. To locate the agmatine binding site and possible catalytic mechanism of CdmS, we compared the crystal structures of HsDHS and BvHSS with a structural model of a CdmS -substrate complex that was constructed using an Alphafold2 prediction model. Based on the differences in substrates and products between CdmS, HsDHS, and BvHSS, it was expected that the structures of CdmS and HsDHS would diverge significantly. However, the substrate-binding site structure as well as the overall structure of CdmS was very similar to the crystal structure of HsDHS and they are largely different from those of BvHSS (Fig. 6 and Supplemental Fig. 3). Figure 6 shows the structure of the substrate-binding site of the complex model of CdmS and agmatine (Fig. 6A), along with those of the HsDHS-spermidine complex (PDB ID 6XXJ) (Fig. 6B)^30^ and the BvHSS-putrescine complex (PDB ID 4TVB) (Fig. 6C)^31^. Most of the aligned residues of CdmS and HsDHS are identical or similar, with two exceptions of residues having different properties (Phe^204^ and Leu^286^ in CdmS vs Asp^243^ and Asp^316^ in HsDHS) (Fig. 6A and B). The structural model of a CdmS suggests that Glu^293^ and Ser^67^ are responsible for the binding to the guanidyl group of agmatine. A conservation of the putative catalytic lysine (Lys^299^ in CdmS, corresponding to Lys^329^ in HsDHS) indicates that the catalytic reaction of CdmS likely proceeds via an enzyme-imine covalent intermediate as has been observed for the HsDHS^32,33^. The model also suggests that Glu^136^ and Asp^143^, which are located at the entrance to the substrate-binding site, are responsible for binding the guanidyl group of a second agmatine substrate. To examine this possibility directly, we are currently investigating the structure of CdmS-substrate complexes using X-ray crystallographic techniques as well as conducting mutagenesis experiments.

## Conclusions

**Figure 7:**
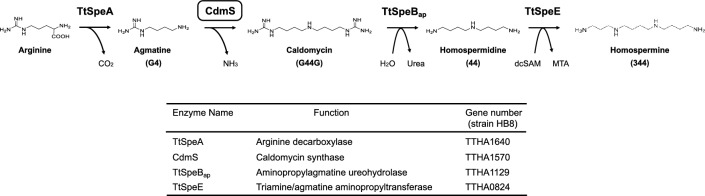
Proposed biosynthetic pathway for homospermidine and homospermine in *T. thermophilus.*

We identified a novel agmatine homocoupling enzyme, CdmS, which is involved in homospermidine biosynthesis in an extreme thermophile *T. thermophilus*, and found that the reaction mechanism was different from those of existing homospermidine synthase enzymes. The CdmS enzyme catalyzes a conjugation of two molecules of agmatine, which produces a diamidino precursor of homospermidine—1,9-bis(guanidino)-5-aza-nonane; caldomycin. Based on these results, we conclude that a new homospermidine biosynthetic pathway exists in *T. thermophilus*. As shown in Fig. 7, homospermidine (44) is produced from agmatine by CdmS in combination with ureohydrolase TtSpeB_ap_. In addition, homospermidine is converted to homospermine (344) by the addition of an aminopropyl group by TtSpeE/TAAPT from decarboxylated *S*-adenosylmethionine produced by TtSpeD2/SAMDC^23^.

## Materials and methods

### Bacterial strains and culture conditions

*T. thermophilus* HB8 (the wild-type strain) and its disrupted strains (Δ*cdmS*) were cultured overnight at 70 ℃ at 160 rpm in rich media of 0.8% tryptone, 0.4% yeast extract, 0.2% NaCl, 0.35 mM CaCl_2_ and 0.4 mM MgCl_2_, and were grown until A_600_ reached 1.0. Prior to use, the strains were grown in synthetic medium^34^ for 24 h to deplete the cells of carryover polyamines. Then, cell culture started at an A_600_ = 0.05, and growth was monitored at 70 ℃ by measuring A_600_ in synthetic medium.

### Polyamine analysis

The wild-type strain and/or its disrupted strains were cultivated in synthetic medium until A_600_ = 0.5 and then harvested. Cells were disrupted in 15% trichloroacetic acid by sonication, for high performance liquid chromatography (HPLC) (HITACHI, Tokyo, Japan) analysis. The mixture was centrifuged, and the supernatant was used for HPLC analysis. HPLC analysis was carried out as described previously^23^. Protein content was determined using a Bradford Assay kit (Bio-Rad, CA, USA).

### Disruption of *cdmS*

Deletion of the CdmS (*cdmS*: TTHA1570) gene in *T. thermophilus* HB8 was performed as described previously^25^. For construction of the deletion plasmid, the gene encoding kanamycin-resistant gene (*htk*) and both the upstream and downstream genomic regions flanking *cdmS* was amplified by polymerase chain reaction (PCR). Two pairs of primers: P1, 5′-CAAGGTGGTACCGCTTGTCAAC-3′/P2, 5′-CTTCTGCATATGTCTCCTTTCCACGC-3′ and P3, 5′-GTTACTGCAGACTCTTCCGGGAGAAAG-3′/P4, 5′-GACCAGGATCCGCACCCTCTTTC-3′. These DNA fragments were assembled and inserted into *Kpn*I-*Nde*I and *Pst*I-*Bam*HI digested pBluescript II SK^+^ plasmid^25,35^ using the NEBuilder HiFi DNA assembly cloning kit (New England Biolabs, MA, USA) according to the manufacturer’s instructions. The DNA mixture was used for the transformation of *E. coli* JM109, and the plasmid was extracted from the kanamycin-resistant cells. To obtain *cdmS* gene-disrupted strains of *T. thermophilus* HB8, the thermophile host was genetically transformed as described previously^25^.

### Expression and purification of enzymes

To make a *cdmS* expression plasmid (pET21cdmS), the gene was amplified by PCR using *T. thermophilus* HB8 genomic DNA as a template and two primers P5, 5′- AAGGATCCCATATGCAGAAGAAGGAACTCCTCTCTACGCC-3′ and P6, 5′- TTAGAATTCTTGCCTTCAGGCGGGCGCTTTC-3′. The DNA fragment was assembled and inserted into *Nde*I and *Eco*RI digested pET21b plasmid (Novagen). The nucleotide sequence of the plasmid was confirmed with a 3130 Genetic Analyzer (Applied Biosystems, MA, USA). *E. coli* BL21(DE3) pLysS harboring pET21cdmS was grown in LB medium supplemented with 100 μg/ml ampicillin until the A_600_ = 0.4 and then induced with 1 mM isopropyl β-D-1-thiogalactopyranoside (IPTG) at 37 °C. After 4 h, cells were harvested by centrifugation and resuspended in 20 mM Tris–HCl (pH 8.0), 10 mM MgCl_2_ and 1 mM dithiothreitol at one-twentieth the volume of the culture medium. The cells were then disrupted by sonication. After removing the cell debris by centrifugation, the cell extract was heated at 80 °C for 1 h and the denatured protein was removed by centrifugation. Supernatant was applied to a TOYOPEARL Butyl 650 M column (TOSOH), preequilibrated with the buffer A (20 mM Tris–HCl, pH 8.0) containing 0.5 M ammonium sulfate. The column was subsequently washed with same buffer. Elution was then performed using linear gradient of buffer A, which contained a decreasing concentration of ammonium sulfate ranging from 0.5 to 0 M. Purification efficiency was evaluated by SDS-PAGE. The purified CdmS was then dialyzed against 20 mM Tris–HCl (pH 8.0) and stored at 4 °C. TtSpeB_ap_ and TtARG were expressed and purified using the same methods as described previously^26^. Protein content was determined using a Bradford Assay kit (Bio-Rad, CA, USA).

### Enzymatic reactions

The activity of CdmS was determined by measuring the decrease in substrate quantity or the amount of homospermidine formed. The reaction mixture consisting of 50 mM Tris–HCl (pH 8.5), 1 mM NAD^+^, 1 mM substrate (agmatine, diaminopropane, putrescine, norspermidine, and/or spermidine) and purified CdmS in a final volume of 100 µL was incubated at 65 °C for 1 h. The post-reaction manipulations were performed in the same manner as described previously^25^. The products of the enzymatic reaction were analyzed by HPLC as described in “Polyamine analysis”. To determine *K*_m_ and *V*_max_, purified TtSpeB_ap_ and 10 µM MnCl_2_ were added to the aforementioned reaction mixture to measure the formation of homospermidine. The ureohydrolase activity of TtSpeB_ap_ and TtAGR was assayed as described previously^26^. Urea produced by the reaction was measured using a Quantichrome Urea Assay Kit (BioAssay Systems, CA, USA) according to the manufacturer’s instructions^36^.

### Guanidyl derivative analysis

The enzymatic reaction mixture as described above was stopped by 5% trichloroacetic acid. The reaction solution was centrifuged and the supernatant was used for HPLC analysis. The detection of guanidyl derivatives was carried out as described previously, with slight modifications^29^. The chromatographic separation was carried out at 75 °C in a TSKgel Polyaminepak column (4.6 mm I.D. × 5 cm) (TOSOH, Japan). The column was equilibrated with the elution buffer [93 mM trisodium citrate dihydrate, 2 M NaCl, 53 mM HCl, 0.68 mM hexanoic acid, 0.08% Brij35, 20% *N*,*N*-dimethylformamide, 20% methanol; pH 5.1 (adjusted by adding NaOH)]. The eluted guanidyl derivatives were automatically mixed with a detection buffer composed of 3 M potassium hydroxide, 2 mM benzoin, 0.7 M 2-mercaptoethanol, and 20% dimethyl sulfoxide. Fluorescence at 435 nm (excitation wavelength, 325 nm) was monitored.

### Structural model of a complex of CdmS and agmatine

An AlphaFold2 prediction model of a monomeric CdmS (entry ID Q5SI10) was obtained from the UniProt protein knowledgebase website (https://www.uniprot.org) ^37^. An agmatine docking model for CdmS was obtained by superposing the dimeric CdmS model to the crystal structure of the human DHS-GC7 (1-guanidinium-7-aminoheptane) complex (PDB ID 1RQD) with modification of GC7 to agmatine. Figures were prepared using the PyMOL Molecular Graphics System, Version 2.0 Schrödinger, LLC (https://pymol.org/2/).

### Supplementary Information

Supplementary Information.

## Data Availability

All data generated or analysed during this study are included in this published article.
