# Supplementary material for: Caldomycin, a new guanidopolyamine produced by a novel agmatine homocoupling enzyme involved in homospermidine biosynthesis
Source: Sci Rep. 2024 Mar 30;14:7566. doi: 10.1038/s41598-024-58296-0 (PMC10981699; doi:10.1038/s41598-024-58296-0)
Supplement: Supplementary file 1 — Supplementary Information. [file 41598_2024_58296_MOESM1_ESM.pptx]

## Slide 1
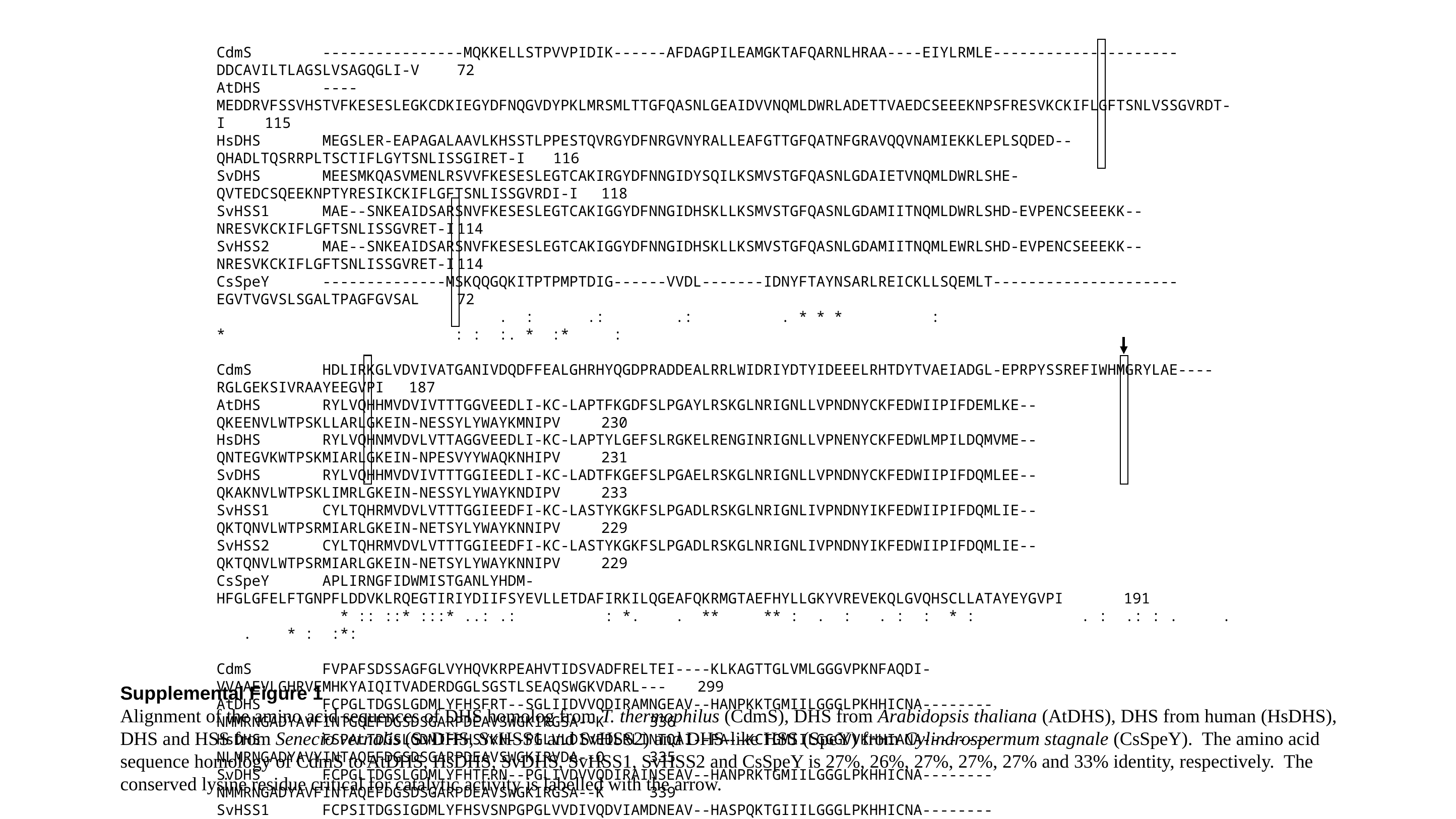

CdmS ----------------MQKKELLSTPVVPIDIK------AFDAGPILEAMGKTAFQARNLHRAA----EIYLRMLE---------------------DDCAVILTLAGSLVSAGQGLI-V	72
AtDHS ----MEDDRVFSSVHSTVFKESESLEGKCDKIEGYDFNQGVDYPKLMRSMLTTGFQASNLGEAIDVVNQMLDWRLADETTVAEDCSEEEKNPSFRESVKCKIFLGFTSNLVSSGVRDT-I	115
HsDHS MEGSLER-EAPAGALAAVLKHSSTLPPESTQVRGYDFNRGVNYRALLEAFGTTGFQATNFGRAVQQVNAMIEKKLEPLSQDED--QHADLTQSRRPLTSCTIFLGYTSNLISSGIRET-I	116
SvDHS MEESMKQASVMENLRSVVFKESESLEGTCAKIRGYDFNNGIDYSQILKSMVSTGFQASNLGDAIETVNQMLDWRLSHE-QVTEDCSQEEKNPTYRESIKCKIFLGFTSNLISSGVRDI-I	118
SvHSS1 MAE--SNKEAIDSARSNVFKESESLEGTCAKIGGYDFNNGIDHSKLLKSMVSTGFQASNLGDAMIITNQMLDWRLSHD-EVPENCSEEEKK--NRESVKCKIFLGFTSNLISSGVRET-I	114
SvHSS2 MAE--SNKEAIDSARSNVFKESESLEGTCAKIGGYDFNNGIDHSKLLKSMVSTGFQASNLGDAMIITNQMLEWRLSHD-EVPENCSEEEKK--NRESVKCKIFLGFTSNLISSGVRET-I	114
CsSpeY --------------MSKQQGQKITPTPMPTDIG------VVDL-------IDNYFTAYNSARLREICKLLSQEMLT---------------------EGVTVGVSLSGALTPAGFGVSAL	72
 . : .: .: . * * * : * : : :. * :* :
CdmS HDLIRKGLVDVIVATGANIVDQDFFEALGHRHYQGDPRADDEALRRLWIDRIYDTYIDEEELRHTDYTVAEIADGL-EPRPYSSREFIWHMGRYLAE----RGLGEKSIVRAAYEEGVPI	187
AtDHS RYLVQHHMVDVIVTTTGGVEEDLI-KC-LAPTFKGDFSLPGAYLRSKGLNRIGNLLVPNDNYCKFEDWIIPIFDEMLKE--QKEENVLWTPSKLLARLGKEIN-NESSYLYWAYKMNIPV	230
HsDHS RYLVQHNMVDVLVTTAGGVEEDLI-KC-LAPTYLGEFSLRGKELRENGINRIGNLLVPNENYCKFEDWLMPILDQMVME--QNTEGVKWTPSKMIARLGKEIN-NPESVYYWAQKNHIPV	231
SvDHS RYLVQHHMVDVIVTTTGGIEEDLI-KC-LADTFKGEFSLPGAELRSKGLNRIGNLLVPNDNYCKFEDWIIPIFDQMLEE--QKAKNVLWTPSKLIMRLGKEIN-NESSYLYWAYKNDIPV	233
SvHSS1 CYLTQHRMVDVLVTTTGGIEEDFI-KC-LASTYKGKFSLPGADLRSKGLNRIGNLIVPNDNYIKFEDWIIPIFDQMLIE--QKTQNVLWTPSRMIARLGKEIN-NETSYLYWAYKNNIPV	229
SvHSS2 CYLTQHRMVDVLVTTTGGIEEDFI-KC-LASTYKGKFSLPGADLRSKGLNRIGNLIVPNDNYIKFEDWIIPIFDQMLIE--QKTQNVLWTPSRMIARLGKEIN-NETSYLYWAYKNNIPV	229
CsSpeY APLIRNGFIDWMISTGANLYHDM-HFGLGFELFTGNPFLDDVKLRQEGTIRIYDIIFSYEVLLETDAFIRKILQGEAFQKRMGTAEFHYLLGKYVREVEKQLGVQHSCLLATAYEYGVPI	191
 * :: ::* :::* ..: .: : *. . ** ** : . : . : : * : . : .: : . . . * : :*:
CdmS FVPAFSDSSAGFGLVYHQVKRPEAHVTIDSVADFRELTEI----KLKAGTTGLVMLGGGVPKNFAQDI-VVAAEVLGHRVEMHKYAIQITVADERDGGLSGSTLSEAQSWGKVDARL---	299
AtDHS FCPGLTDGSLGDMLYFHSFRT--SGLIIDVVQDIRAMNGEAV--HANPKKTGMIILGGGLPKHHICNA--------NMMRNGADYAVFINTGQEFDGSDSGARPDEAVSWGKIRGSA--K	336
HsDHS FSPALTDGSLGDMIFFHSYKN--PGLVLDIVEDLRLINTQAI--FA--KCTGMIILGGGVVKHHIANA--------NLMRNGADYAVYINTAQEFDGSDSGARPDEAVSWGKIRVDA--Q	335
SvDHS FCPGLTDGSLGDMLYFHTFRN--PGLIVDVVQDIRAINSEAV--HANPRKTGMIILGGGLPKHHICNA--------NMMRNGADYAVFINTAQEFDGSDSGARPDEAVSWGKIRGSA--K	339
SvHSS1 FCPSITDGSIGDMLYFHSVSNPGPGLVVDIVQDVIAMDNEAV--HASPQKTGIIILGGGLPKHHICNA--------NMMRNGADFAVFINTAQEYDGSDSGARPDEAVSWGKISSTG--K	337
SvHSS2 FCPSITDGSIGDMLYFHSVSNPGPGLVVDIVQDVIAMDNEAV--HASPQKTGIIILGGGLPKHHICNA--------NMMRNGADFAVFINTAQEYDGSDSGARPDEAVSWGKISSTG--K	337
CsSpeY YTSSPGDSSIGMNVAALALEG--SQLVIDPSIDVNETAAIAYGARESEGKSAAVIIGGGSPKNFLLQTQPQIHEVLGLEERGHDYFIQFTDARPDTGGLSGATPAEAVSWGKIDPLELPN	309
 : . *.* * : : :* *. :. :::*** *:. : . . .: : :. . *. **: ** ****:
CdmS SQMVFAEATLAFPLLASYVWHRAPARAKRRYAEL----FR----EK-----APA--------------------	340
AtDHS TVKVYCDATIAFPLLVAETFATKRDQTCESKT------------------------------------------	368
HsDHS PVKVYADASLVFPLLVAETFAQKMDAFMHEKN------ED----------------------------------	369
SvDHS SVKVHCDATIAFPLLVAETFAAKREQSAEPSS------------------------------------------	371
SvHSS1 AVKVHCDATIAFPLLVAETFAVKKEKASKVNG------F-----------------------------------	370
SvHSS2 AVKVHCDATIAFPLLVAETFAVKKEKASKVNG------F-----------------------------------	370
CsSpeY TIVCYTDSTIALPLVTAYVMNQCQPRPLKRLYDQREALYDKLQKDYLAAKDKPSVADGKS EEVATYPCGTPVKR	383
 . ::::.:**:.: . .
Supplemental Figure 1
Alignment of the amino acid sequences of DHS homolog from T. thermophilus (CdmS), DHS from Arabidopsis thaliana (AtDHS), DHS from human (HsDHS), DHS and HSS from Senecio vernalis (SvDHS, SvHSS1 and SvHSS2) and DHS-like HSS (SpeY) from Cylindrospermum stagnale (CsSpeY). The amino acid sequence homology of CdmS to AtDHS, HsDHS, SvDHS, SvHSS1, SvHSS2 and CsSpeY is 27%, 26%, 27%, 27%, 27% and 33% identity, respectively. The conserved lysine residue critical for catalytic activity is labelled with the arrow.

## Slide 2
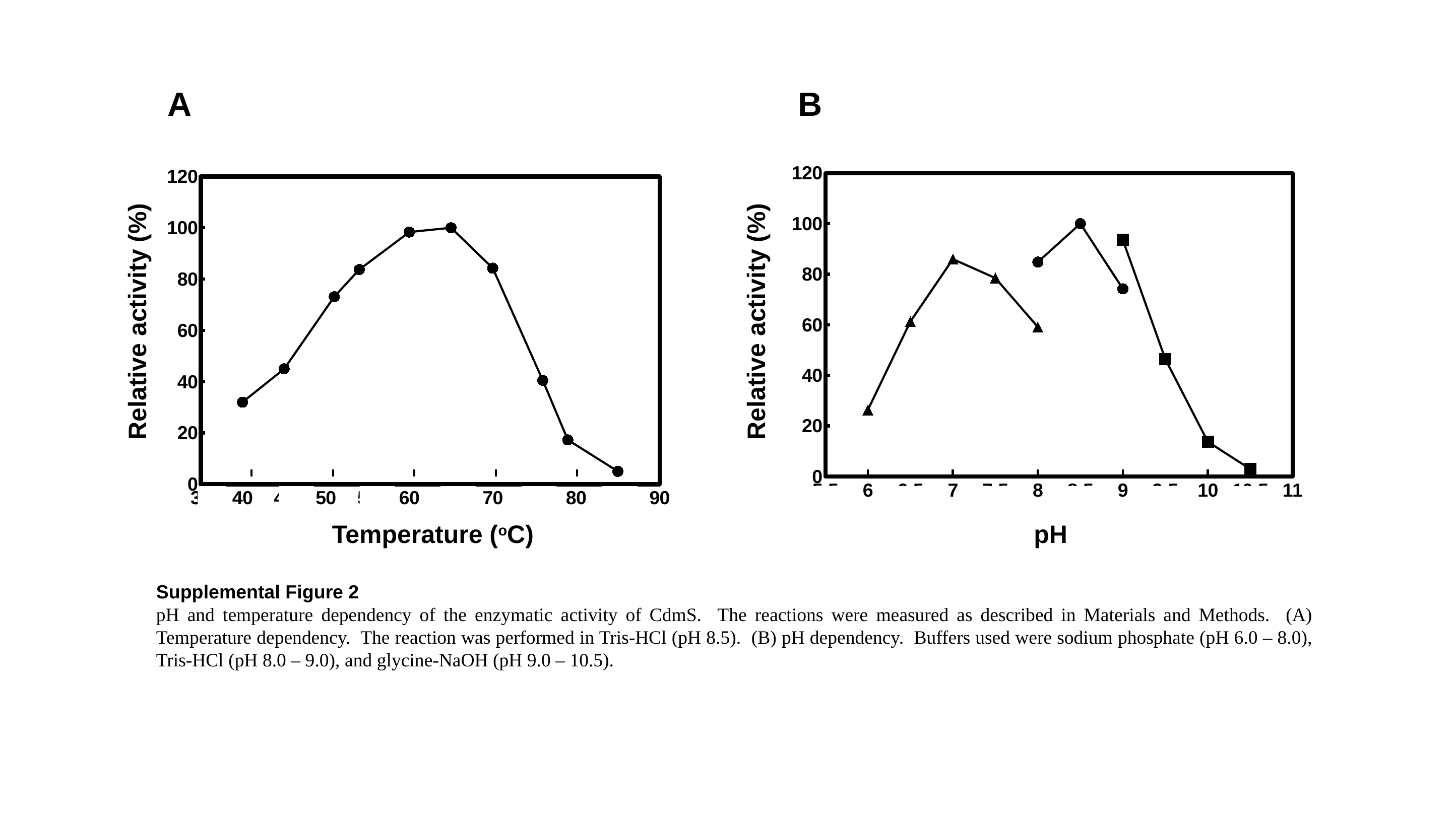

A
B
### Chart
| Category | Phosphate buffer | Tris buffer | Glycine buffer |
|---|---|---|---|
### Chart
| Category | |
|---|---|Relative activity (%)
Relative activity (%)
Temperature (oC)
pH
Supplemental Figure 2
pH and temperature dependency of the enzymatic activity of CdmS. The reactions were measured as described in Materials and Methods. (A) Temperature dependency. The reaction was performed in Tris-HCl (pH 8.5). (B) pH dependency. Buffers used were sodium phosphate (pH 6.0 – 8.0), Tris-HCl (pH 8.0 – 9.0), and glycine-NaOH (pH 9.0 – 10.5).

## Slide 3
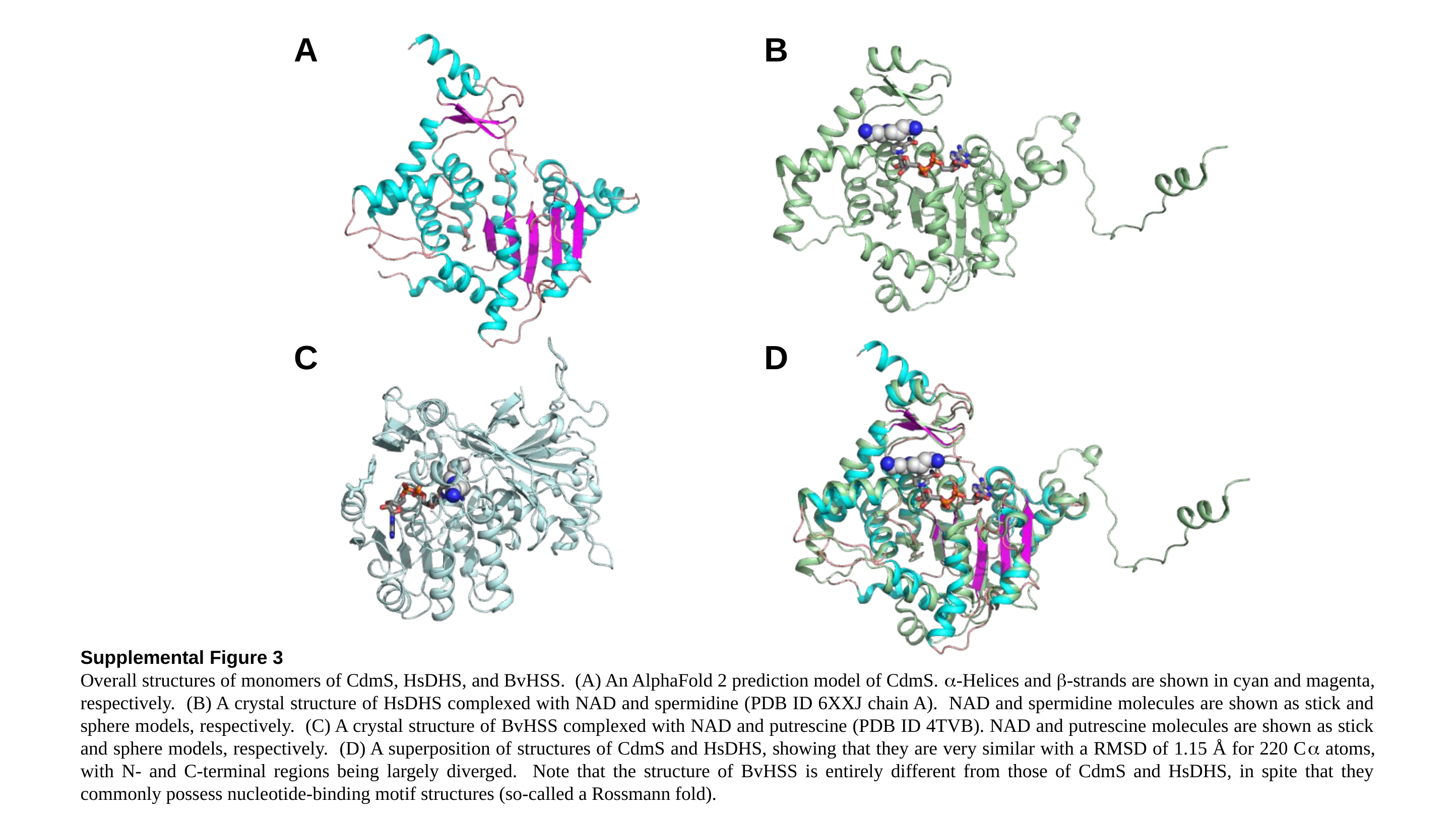

A
B
C
D
Supplemental Figure 3
Overall structures of monomers of CdmS, HsDHS, and BvHSS. (A) An AlphaFold 2 prediction model of CdmS. a-Helices and b-strands are shown in cyan and magenta, respectively. (B) A crystal structure of HsDHS complexed with NAD and spermidine (PDB ID 6XXJ chain A). NAD and spermidine molecules are shown as stick and sphere models, respectively. (C) A crystal structure of BvHSS complexed with NAD and putrescine (PDB ID 4TVB). NAD and putrescine molecules are shown as stick and sphere models, respectively. (D) A superposition of structures of CdmS and HsDHS, showing that they are very similar with a RMSD of 1.15 Å for 220 Ca atoms, with N- and C-terminal regions being largely diverged. Note that the structure of BvHSS is entirely different from those of CdmS and HsDHS, in spite that they commonly possess nucleotide-binding motif structures (so-called a Rossmann fold).

## Slide 4
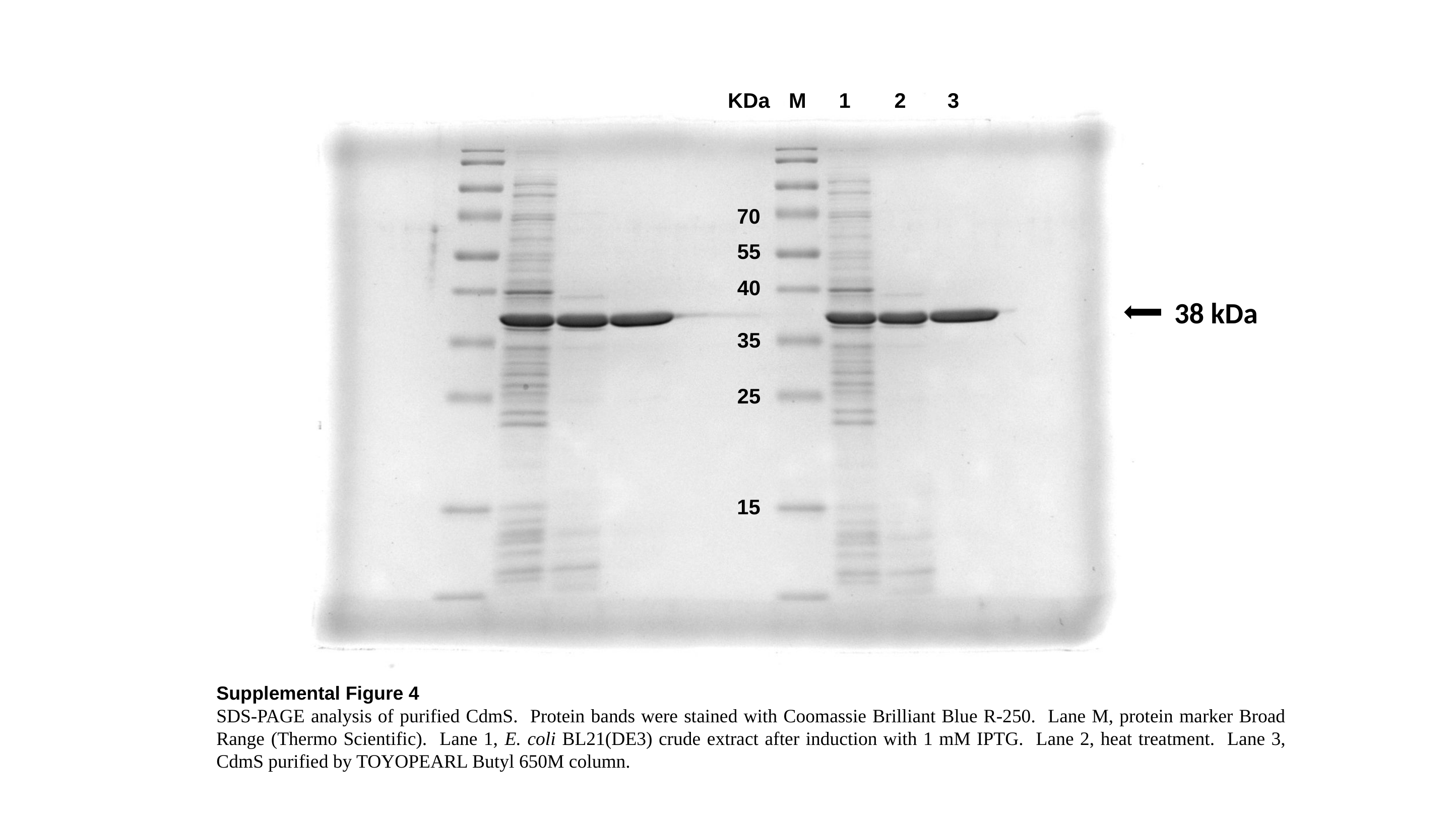

KDa
M
1
2
3
70
55
40
38 kDa
35
25
15
Supplemental Figure 4
SDS-PAGE analysis of purified CdmS. Protein bands were stained with Coomassie Brilliant Blue R-250. Lane M, protein marker Broad Range (Thermo Scientific). Lane 1, E. coli BL21(DE3) crude extract after induction with 1 mM IPTG. Lane 2, heat treatment. Lane 3, CdmS purified by TOYOPEARL Butyl 650M column.
